# Supplementary material for: Thermal Hall effects due to topological spin fluctuations in YMnO3
Source: Nat Commun. 2024 Jan 4;15:243. doi: 10.1038/s41467-023-44448-9 (PMC10764330; doi:10.1038/s41467-023-44448-9)
Supplement: Supplementary file 1 — Supplementary Information [file 41467_2023_44448_MOESM1_ESM.pdf]

Supplementary Information: Thermal Hall effects due to topological spin fluctuations in $YMnO_3$

Ha-Leem Kim[1,2], Takuma Saito[3], Heejun Yang[1,2], Hiroaki Ishizuka[4], Matthew John Coak[1,2,5], Jun Han Lee[6], Hasung Sim[1,2], Yoon Seok Oh[6], Naoto Nagaosa[3,7*], Je-Geun Park[1,2,8*]

[1]Center for Quantum Materials & Department of Physics and Astronomy, Seoul National University, Seoul 08826, Republic of Korea
[2]Center for Correlated Electron Systems, Institute for Basic Science, Seoul 08826, Republic of Korea
[3]Department of Applied Physics, The University of Tokyo, Bunkyo-ku, Tokyo, 113-8656, Japan
[4]Department of Physics, Tokyo Institute of Technology, Meguro-ku, Tokyo, 152-8551, Japan
[5]Department of Physics, University of Warwick, Coventry CV4 7AL, United Kingdom
[6]Department of Physics, Ulsan National Institute of Science and Technology, Ulsan 44919, Republic of Korea
[7]RIKEN Center for Emergent Matter Science (CEMS), Wako, Saitama 351-0198, Japan
[8]Institute of Applied Physics, Seoul National University, Seoul 08826, Republic of Korea

These authors contributed equally: Ha-Leem Kim, Takuma Saito and Heejun Yang
*Corresponding author's e-mail: nagaosa@riken.jp & jgpark10@snu.ac.kr

Supplementary Note 1: Magnetic field-dependent thermal Hall effect

A single crystal of $YMnO_3$ was aligned to have the c-axis perpendicular to the sample plane and cut into a bar shape. Distance between leads measuring ΔT_x and ΔT_y were ~1.91 and ~2.05 mm, respectively for sample1, and ~1.75, ~1.62 mm for sample 2. The magnetic field was applied parallel to the crystalline c-axis. Thermal Hall effect (THE) measurements were conducted using a home-built thermal Hall probe and a separate electronic measurement system. The constructed thermal Hall measurement puck with the mounted sample, thermometers and heater is shown in Supplementary Fig. 1. The use of three capacitive thermometers made it possible to achieve ultra-high-resolution temperature measurements under a high magnetic field without lengthy field calibrations. This setup and the advances in instrumentation have allowed us to measure far smaller thermal Hall signals than previously resolvable over a much wider temperature range and to investigate materials not possible to study previously. Details of the experimental method, the stability of temperature, resolution, and self-heating of the thermometers are given in our previous work[1].

As mentioned in the main text, the applied magnetic field was swept from positive to negative values before backing to the positive value to avoid artificial signals induced by temperature drift. An example of the raw data (sample 1) for a single measurement with the field sequence is shown in Supplementary Fig. 2. The temperature reading of all three thermometers responded sharply to the applied magnetic field, demonstrating good thermal links to the sample. Thermometers 2 and 3 measure transverse temperature difference, and the extracted ΔT_y from the raw data is shown in Supplementary Fig. 3a. ΔT_y is different for the positive and negative fields, indicating an observation of THE. Note that ΔT_y is highly

similar for the two different positive magnetic field points, even if they are separated in time by over an hour. This assures us that the thermal drift of ΔT_y is negligible over the measurement time in our setup (the experiment was still conducted using this $+,-,+$ field sequence). Supplementary Fig. 3b shows magnetic field dependent $\Delta T_y^{\mathrm{Anti-sym}}$ extracted from Supplementary Fig. 3a. The signal is linear to the magnetic field but extremely small (smaller than 0.2 mK for the maximum value for sample 1).

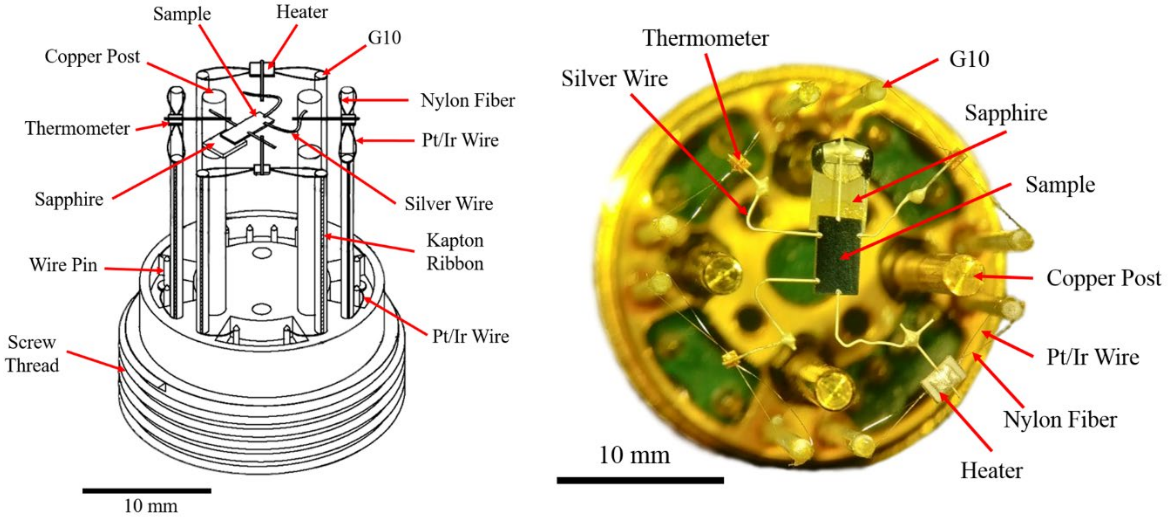

Supplementary Fig. 1 | Details of our thermal Hall measurement setup.

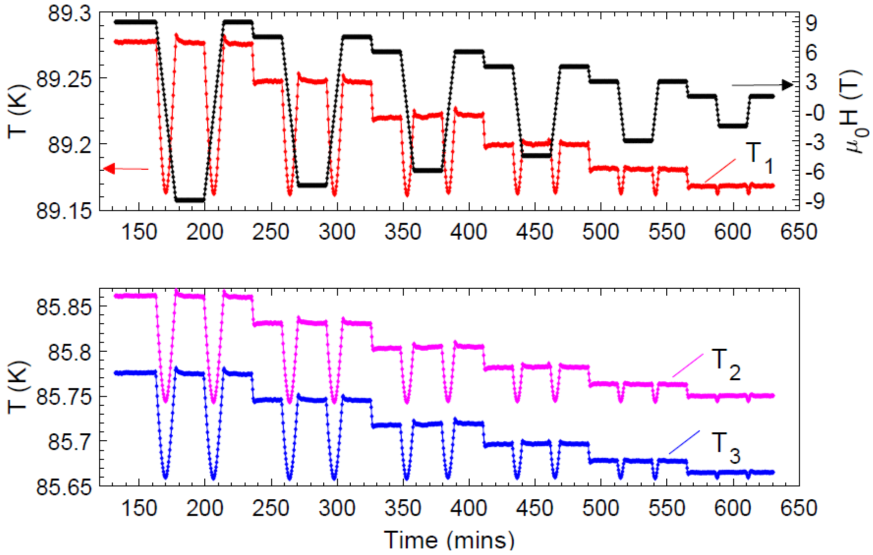

Supplementary Fig. 2 | The raw data of $YMnO_3$ (sample 1) was taken at 87.5 K, with the temperature readings taken from the three thermometers and the magnetic field plotted against time. The longitudinal temperature difference was set to ΔT_x ~ 3.5 K.

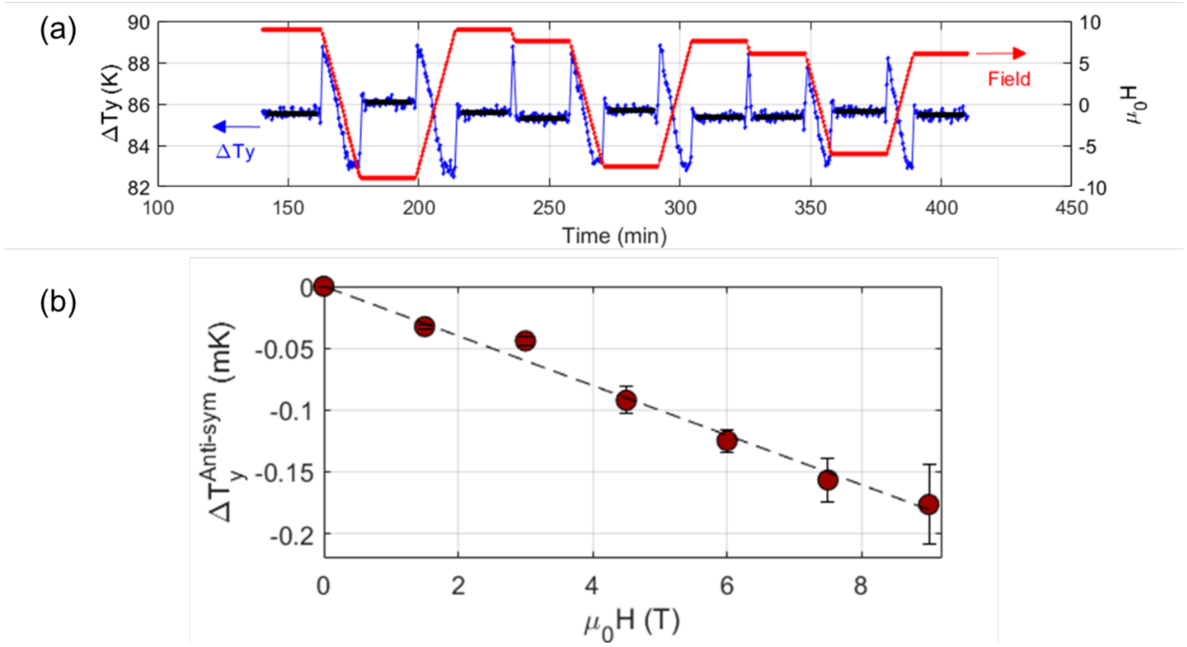

Supplementary Fig. 3 | a ΔT_y and magnetic field values plotted against time (sample 1). ΔT_y is larger for a negative field value compared to a positive one, indicating the existence of a thermal Hall effect. The black bars are constant fits to ΔT_y for a fixed magnetic field. **b** Extracted $\Delta T_y^{\text{Anti-sym}}$ values at 87.5 K, plotted against the applied magnetic field. The longitudinal temperature difference was set to ΔT_x ~ 3.5 K. Minimal thermometer self-heating of ~ 0.04 nW was maintained. Error bars are standard deviation which were obtained by multiple measurements.

Supplementary Note 2: Analysis of thermal Hall angle ratio, κ_{xy}/κ_{xx}

As shown in the Fig. 2a of the main text, around T_{N}, phonon scattering from strong spin-fluctuation arising from geometrical frustration leads to significant suppression of κ_{xx}[2]. Also, the thermal Hall angle ratio (THAR), κ_{xy}/κ_{xx}, exhibits a maximal value at T_{N}, where spin-fluctuation are strongest and decrease in magnitude at temperature away from T_{N}. Given the observation, one might expect the observed THE is due to phonons generated by phonon's skew scattering from strong spin fluctuation. However, κ_{xx}, which is in the denominator of THAR, has a huge local minimum at T_{N}. Thus, within the wide variation of the temperature dependence of κ_{xy}, THAR will always have a maximal value of around T_{N}. In other words, the experimental temperature dependence of κ_{xy}/κ_{xx} is highly dominated by the abnormal temperature dependence of κ_{xx}, misleading one to expect a phonon skew scattering

mechanism. To see unperturbed THAR, we calculated modified THAR, $\kappa_{xy}/\kappa_{xx}^{\mathrm{ph-fit}}$, where $\kappa_{xx}^{\mathrm{ph-fit}}$ is phonon only fit the experimental κ_{xx} assuming scattering between phonons and spin fluctuation are absent.

Phonon fits the experimental κ_{xx}, heat flowing along the crystalline c-axis, was done by Sharma et al.[2]. We outline here a phonon fit to the ab plane κ_{xx} using a similar method. The formula for phonon thermal conductivity is given as Supplementary Eq. (1), the Debye-Callaway model[3]. τ^{-1}, v_s, Θ_D are the phonon scattering rate, sound velocity, and the Debye temperature respectively (Θ_D=290 K, $v_s = 2250$ m/s for $YMnO_3$[2]). The scattering rate, τ^{-1} is given as Eq.S2, where the individual terms correspond to the effect of dislocation, point defect, phonon-phonon scattering, and boundary scattering[2]. b and L correspond to the number of atoms in the unit cell and the scattering boundary.

The values of the free-fitting parameters $A_1(=20\times 10^{-5})$ and $L(=$ 0.2mm) were taken as the same values used for the c-axis as done by Sharma et al.[2], and the fitting parameter A_2 and A_3 was tuned to $17\times 10^{-43}\,\mathrm{s}^3$ and $5.5\times 10^{-18}\,\mathrm{sK}^{-1}$ respectively. Note that this value is consistent with the sample prepared using the same method[3].

$$\kappa_{\mathrm{ph}} = \frac{k_B}{2\pi^2 v_s}\left(\frac{k_{\mathrm{B}}}{\hbar}\right)^3 T^3 \int_0^{\frac{\Theta_D}{T}} \frac{x^4 e^x}{(e^x-1)^2}\tau(\omega,T)dx, \tag{1}$$

$$\tau^{-1} = A_1\omega + A_2\omega^4 + A_3 T\omega^2 \exp\left(-\frac{\Theta_D}{bk_{\mathrm{B}}T}\right) + \frac{v_s}{L}, \tag{2}$$

Plotting $\kappa_{xy}/\kappa_{xx}^{\mathrm{ph-fit}}$ (see Supplementary Fig. 4), we can see a considerable change in temperature dependence compared to the original κ_{xy}/κ_{xx} (we do not see any signature of phonon skew scattering induced THE). The conclusion remains unchanged under a wide variation of fitting parameters. We could go further and compare the suppression degree of κ_{xx}, parametrized as $\left(\kappa_{xx}^{\mathrm{ph-fit}} - \kappa_{xx}^{\mathrm{exp}}\right)\times \text{scaling factor}$, with $\kappa_{xy}/\kappa_{xx}^{\mathrm{ph-fit}}$. As shown in Supplementary Fig. 4, two parameters seem irrelevant, indicating the phonon skew scattering scenario's false reasoning.

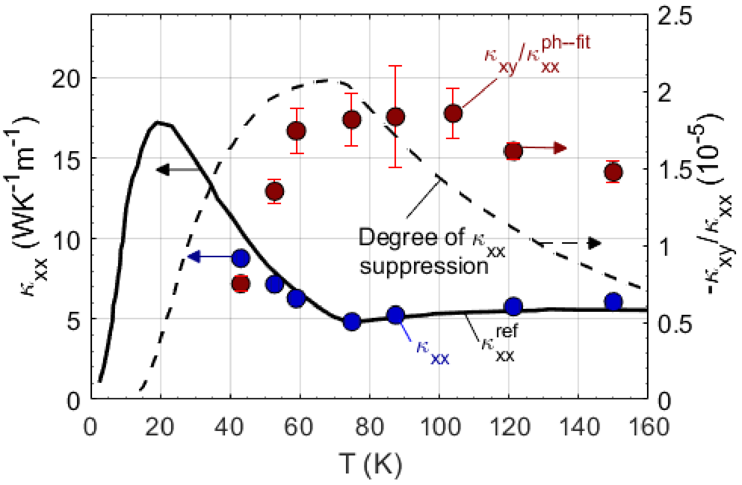

Supplementary Fig. 4 | Modified temperature-dependent thermal Hall angle ratio, $\kappa_{xy}/\kappa_{xx}^{\mathrm{ph-fit}}$, at 9 T (red circle, right axis), and thermal conductivity at 0 T (blue circle, left axis). Zero field reference thermal conductivity data from the Supplementary Reference[2] is also drawn (left axis). The dashed line is annotated as Degree of κ_{xx} suppression is the difference between phonon-only fit and experimental value of thermal conductivity, multiplied with a proper scaling factor (right axis); $(\kappa_{xx}^{\mathrm{ph-fit}} - \kappa_{xx}^{\mathrm{exp}}) \times \text{scaling factor}$. Error bars are standard deviation which were obtained by multiple measurements.

Supplementary Note 3: Theory for thermal Hall conductivity

We can apply the Kubo formula to the thermal transport coefficients like the electric transport theory. The thermal conductance tensor is defined by $\langle J^{E,\mu}\rangle = -\kappa_{\mu\nu}^{\mathrm{Kubo}}\partial_\nu T$, where $\kappa_{\mu\nu}^{\mathrm{Kubo}}$ is calculated by

$$\kappa_{\mu\nu}^{\mathrm{Kubo}} = -\lim_{\omega\to 0}\frac{C_{\mu\nu}^R(\omega) - C_{\mu\nu}^R(0)}{i\omega}, \qquad C_{\mu\nu}(i\omega) = -\int_0^\beta d\tau\; e^{i\omega\tau}\langle T_\tau J^{E,\mu}(\tau)J^{E,\nu}(0)\rangle. \tag{3}$$

Here, $C_{\mu\nu}^R(\omega)$ is the retarded correlation function of energy current. Supplementary Eq. 3 is equivalent to

$$\kappa_{\mu\nu}^{\mathrm{Kubo}} = \frac{1}{k_B T^2}\int_0^\infty dt\,\langle J^{E,\mu}(t); J^{E,\nu}(0)\rangle, \tag{4}$$

where $\langle A;B\rangle$ is the Kubo correlation function, which is defined by $\langle A;B\rangle = \beta^{-1}\int_0^\beta d\tau\, A(-i\tau)B(0)$. In the classical limit, the Kubo pairing reduces to the thermal average of the product: $\langle A;B\rangle \xrightarrow{\text{classical}} \langle AB\rangle = \mathrm{Tr}\big(e^{-\beta H}AB\big)/\mathrm{Tr}\big(e^{-\beta H}\big)$.

It is known that $\kappa_{xy}^{\mathrm{Kubo}}$ does not give the net thermal Hall conductance; it includes a $1/T$ term, which diverges at low temperatures. We must consider an additional term related

to the so-called energy magnetization (EM) to cancel the divergent term. The formula for the EM, which we denote by $\mathbf{M}^E$, was given by Qin *et al.*[4] in the form of a differential equation:

$$-\partial_T\left(\frac{\mathbf{M}^E}{T^2}\right)=\frac{\mu^{E,z}}{T^3}, \qquad \boldsymbol{\mu}^E:=\frac{1}{2iT}\nabla_q\times\langle H_{-\mathbf{q}};\mathbf{J}_{\mathbf{q}}^E\rangle_{\mathrm{eq}}\big|_{\mathbf{q}\to 0}. \tag{5}$$

Here, $H_{-\mathbf{q}}=\sum_i e^{-i\mathbf{q}\cdot\mathbf{r}_i}H_i$ with H_i being the Hamiltonian (or energy) at i-th site, and $\mathbf{J}_{\mathbf{q}}^E$ as well. The EM is obtained by integrating Supplementary Eq. 5 over T. Then we have to set a physically proper initial condition to fix the value of $\mathbf{M}^E$; here, we assume $\lim_{T\to\infty}\mathbf{M}^E=0$ and calculate

$$M^{E,z}(T)=T^2\int_T^{\infty}\frac{\mu^{E,z}}{T'^3}dT'. \tag{6}$$

The correction term to κ_{xy} reads $\kappa_{xy}^{\mathrm{EM}}=2M^{E,z}/(VT)$, where V is the volume of the system, and the net thermal Hall conductance is $\kappa_{xy}=\kappa_{xy}^{\mathrm{Kubo}}+\kappa_{xy}^{\mathrm{EM}}$.

Supplementary Note 4: Definition and formula of the energy current

In the lattice model, the Hamiltonian can be decomposed into local Hamiltonians by $H=\sum_i H_i$, and the conservation law of energy reads

$$\partial_t H_i+\sum_j j_{ji}^E=0. \tag{7}$$

Here, j_{ji}^E is defined at each bond and represents the energy flowing out of i-th site into j-th site per unit of time. The total energy current is given by the summation of j_{ji}^E weighted by site distance: $\mathbf{J}^E=\frac{1}{2}\sum_{i,j}j_{ji}^E\mathbf{r}_{ji}$ with $\mathbf{r}_{ji}=\mathbf{r}_j-\mathbf{r}_i$. By distributing $\mathbf{J}^E$ to each site, we obtain the energy current density $\mathbf{j}_i^E=\frac{1}{2}\sum_j j_{ji}^E\mathbf{r}_{ji}$. It is shown that there is no net energy current at thermal equilibrium by utilizing Bloch's theorem[5]: $\langle\mathbf{J}^E\rangle_{\mathrm{eq}}=0$. It should be noted that there is ambiguity in adding a divergence-free energy current. This is expressed by $j_{ij}^{E,\mathrm{rot}}=\sum_k A_{ijk}$, where A_{ijk} is anti-symmetric for the exchange of any pairs in $\{i,j,k\}$. $\sum_j j_{ij}^{E,\mathrm{rot}}=\sum_{jk}A_{ijk}=\sum_{jk}A_{ikj}=-\sum_{jk}A_{ijk}=0$. One solution for j_{ji}^E, which satisfies Eq. S7, is $j_{ji}^E=\frac{i}{\hbar}\left[H_i,H_j\right]$. However, in our case, it is more convenient to directly calculate $\partial_t H_i$. The result is

$$
j_{i\to j}^{E} = \frac{1}{\hbar}\Bigg[(\mathbf{h} - 2\Delta \mathbf{S}_i)\cdot\left(J_{ij}(\mathbf{S}_i\times\mathbf{S}_j) + \frac{1}{2}\left(\mathbf{S}_i\times(\mathbf{S}_j\times\mathbf{D}_{ij}) + \mathbf{S}_j\times(\mathbf{S}_i\times\mathbf{D}_{ij})\right)\right)
+ \frac{1}{2}\sum_k \Big[J_{ik}J_{kj}\mathbf{S}_i\cdot(\mathbf{S}_k\times\mathbf{S}_j) + (\mathbf{S}_i\times\mathbf{D}_{ik})\cdot\left(\mathbf{S}_k\times(\mathbf{S}_j\times\mathbf{D}_{kj})\right) + J_{ik}(\mathbf{S}_i\times\mathbf{S}_k)
\cdot(\mathbf{S}_j\times\mathbf{D}_{kj}) - J_{kj}(\mathbf{S}_i\times\mathbf{D}_{ik})\cdot(\mathbf{S}_k\times\mathbf{S}_j)\Big]\Bigg], \tag{8}
$$

which is consistent with the supplementary References[6,7], except that our model includes the anisotropy term.

Supplementary Note 5: Calculation of the energy magnetization

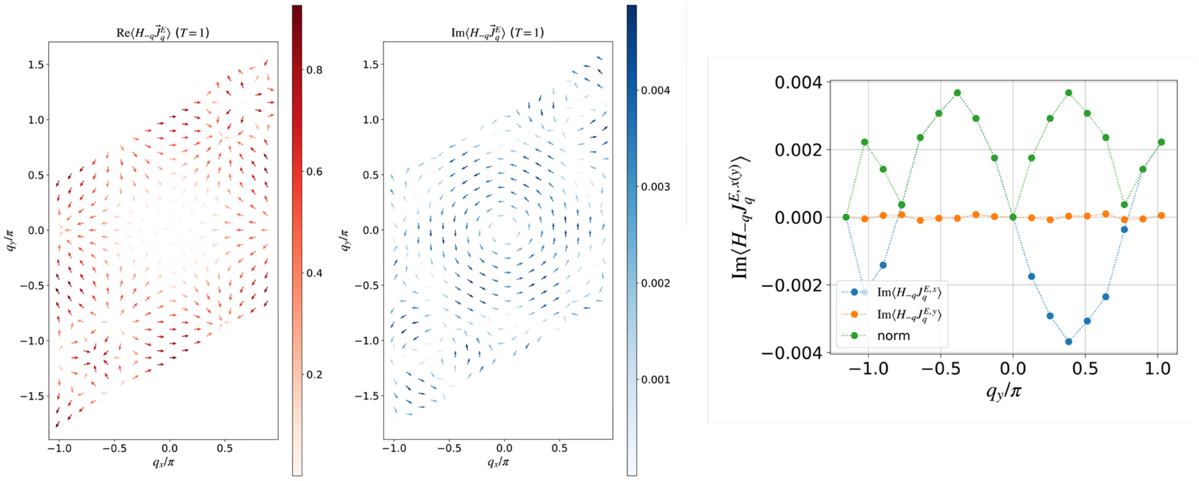

Supplementary Fig. 5 | Left: Plot of the real and imaginary parts of $\langle H_{-\mathbf{q}} J_{\mathbf{q}}^{E}\rangle$ in the Brillouin zone, where $T = J_1$ and lattice size $N = 18\times 18$. One can see the vortex structure in the imaginary part. Right: The plot of the imaginary part along the $q_x = 0$ line, from which one can determine q_{cut}.

Here, we show the numerical results of $\mathbf{M}^E$. In the previous paper by Carnahan *et al.*[7], the energy magnetization was calculated by $\mathbf{M}^E = \frac{1}{V}\sum_i \mathbf{r}_i \times \mathbf{J}_i^E$ under the open boundary condition. However, while their method is helpful in the case of the square lattice, we found it does not work in the triangular lattice; the result depends on the choice of lattice configuration. For this reason, we instead used Supplementary Eq. 6, which requires a derivative in the Fourier space. Seemingly, the derivative can be obtained from data at small $|\mathbf{q}|$, but it fails since the fluctuation of $\langle H_{-\mathbf{q}} J_{\mathbf{q}}^{E}\rangle$ is significant in the vicinity of $\mathbf{q} = 0$. Therefore, we fitted the configuration of $\mathrm{Im}\,\langle H_{-\mathbf{q}} J_{\mathbf{q}}^{E}\rangle$ by circulating vectors $\mathbf{v}_{\mathrm{fit}} = [-p_0 q_y, p_1 q_x]$, where p_0 and p_1 are

the fitting parameters. Supplementary Fig. 5 shows the vector plots of $\langle H_{-\mathbf{q}}\mathbf{J}^E_{\mathbf{q}}\rangle$ at $T = J_1$. One can see a vortex structure in the imaginary part, which gives rise to $\boldsymbol{\mu}^E$. The numerical result for $\boldsymbol{\mu}^E$ is shown in the left panel of Supplementary Fig. 6. In this calculation, we performed 2×10^4 MC sweeps for equilibration and 10^5 MC sweeps for measurement on $N = 18\times 18$ lattice. Rotation of $\boldsymbol{\mu}^E$ can be obtained by fitting the vortex structure with a function $\mathbf{v}_{\rm fit} = [-p_0 q_y, p_1 q_x]$ in a circle $|\mathbf{q}| < q_{\rm cut}$. The cutoff $q_{\rm cut}$ is determined so that $\langle H_{-\mathbf{q}}; \mathbf{J}^E_{\mathbf{q}}\rangle$ is linear in q_y. From the right panel of Supplementary Fig. 5, we determined the cutoff to be $q_{\rm cut}/\pi \sim 0.2$.

The energy magnetization is obtained by the integral Supplementary Eq. 6, where the $T\to\infty$ limit cannot be achieved by numerical simulation. Instead, we analyzed the asymptotic behaviour of $\boldsymbol{\mu}^E$ by high-T expansion:

$$\langle H_{-\mathbf{q}} J^E_{\mathbf{q}}\rangle = \sum_{i,k} e^{i\mathbf{q}\cdot(\mathbf{r}_i-\mathbf{r}_k)} \frac{1}{\mathrm{Tr}(e^{-\beta H})}\mathrm{Tr}\left(e^{-\beta H} H_i J^E_k\right)$$

$$= \sum_{i,k} e^{i\mathbf{q}\cdot(\mathbf{r}_i-\mathbf{r}_k)}(1+\beta \mathrm{Tr} H + \cdots)\left[\mathrm{Tr} H_i J^E_k - \beta \mathrm{Tr}\left(H H_i J^E_k\right) + \cdots\right]$$

$$= \sum_{i,k} e^{i\mathbf{q}\cdot(\mathbf{r}_i-\mathbf{r}_k)}\left[\mathrm{Tr}\left(H_i J^E_k\right) + \beta\left(\mathrm{Tr}(H)\mathrm{Tr}\left(H_i J^E_k\right) - \mathrm{Tr}\left(H H_i J^E_k\right)\right) + \mathcal{O}(\beta^2)\right]. \quad \text{(S9)}$$

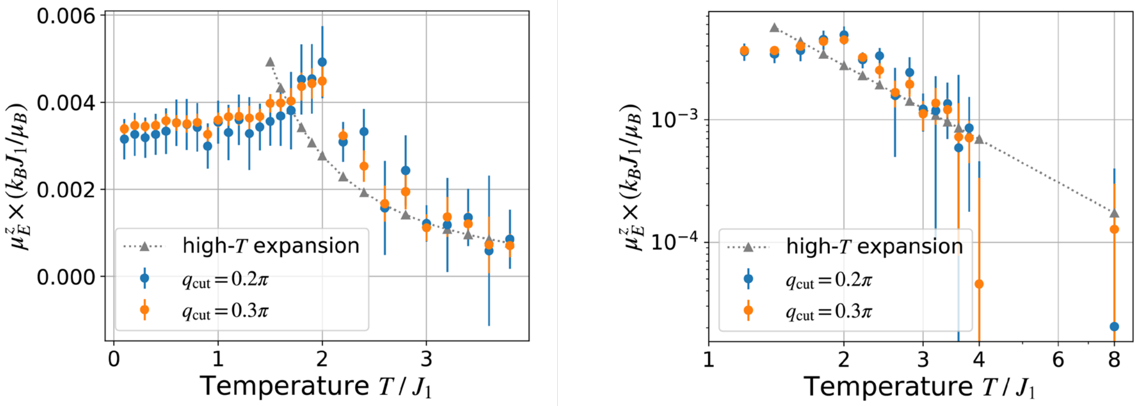

Supplementary Fig. 6 | Temperature dependence of $\mu^{E,z}$. The dotted grey line shows the high-T expansion result, from which one can see that the T^{-2}-law seems to hold for $T \gtrsim 2.6 J_1$. The cutoff is set to $q_{\rm cut} = 0.2\pi$ and 0.3π.

The factors H, H_i, and J^E_k are polynomials of spins. In the classical regime, the trace operator Tr is integral over angular degrees of freedom θ and ϕ, i.e., $\mathrm{Tr}(\cdots) = \frac{1}{4\pi}\int_0^{2\pi} d\phi \int_0^{\pi} d\theta \sin\theta\,(\cdots)$. Then the trace of spins is $\mathrm{Tr}\left(S_i^\mu\right)^n = 0$ for odd n and $S^n/(n+1)$

for even n. By using this, the high-T expansion can be performed. After some algebra, we found that the $\mathcal{O}(\beta^0)$ term in Supplementary Eq. 9 vanishes. This is because the phase factor $e^{i\mathbf{q}\cdot(\mathbf{r}_i-\mathbf{r}_k)}$ is accompanied with $\mathbf{r}_i-\mathbf{r}_k$ in the $\mathcal{O}(\beta^0)$-term and therefore, its rotation is zero, i.e., $\nabla_q\times\left[e^{i\mathbf{q}\cdot(\mathbf{r}_i-\mathbf{r}_k)}(\mathbf{r}_i-\mathbf{r}_k)\right]=-i\nabla_q\times\nabla_q e^{i\mathbf{q}\cdot(\mathbf{r}_i-\mathbf{r}_k)}=0$. Therefore, $\boldsymbol{\mu}^E=\mathcal{O}(\beta^2)$ for $T\gg J_1$, which means

$$\mathbf{M}^E\propto T^2\int_T^\infty dT'\frac{1}{(T')^5}\sim\frac{1}{T^2}\ (T\gg J_1).$$

In the trimerized triangular lattice, the β-linear term is given by

$$\nabla_q\times\langle H_{-\mathbf{q}}J^\mu_{E,\mathbf{q}}\rangle\big|_{\mathbf{q}\to 0}=\frac{\beta}{2\sqrt{3}}S^6hV_{\rm DM}(J_1-J_2)^2. \tag{10}$$

For our parameters, the coefficient is $\frac{\beta}{2\sqrt{3}}S^6hV_{\rm DM}(J_1-J_2)^2=0.01109$ with $h=0.5$. In fact, the numerical result in Supplementary Fig. 6 is consistent with the T^{-2}-law for $T\gtrsim 2.6J_1$, including the coefficient. Thus, we analytically integrate $\mu^{E,z}/T^3$ above $T=3J_1$ by using Supplementary Eq. 10 while we use the numerical data for $T<3J_1$. The resultant $\mathbf{M}^E$, together with $\kappa_{xy}^{\rm EM}$, is shown in Supplementary Fig. 7. Since the mesh for integral is too large at low temperatures, we assumed that $\mu^{E,z}$ is constant for $T\le 0.5J_1$ and numerically performed the integral in that region. As a result, we have $\kappa_{xy}^{\rm EM}\propto T^{-1}$ in the $T\to 0$ limit, which is supposed to cancel the T^{-1} divergence of $\kappa_{xy}^{\rm Kubo}$.

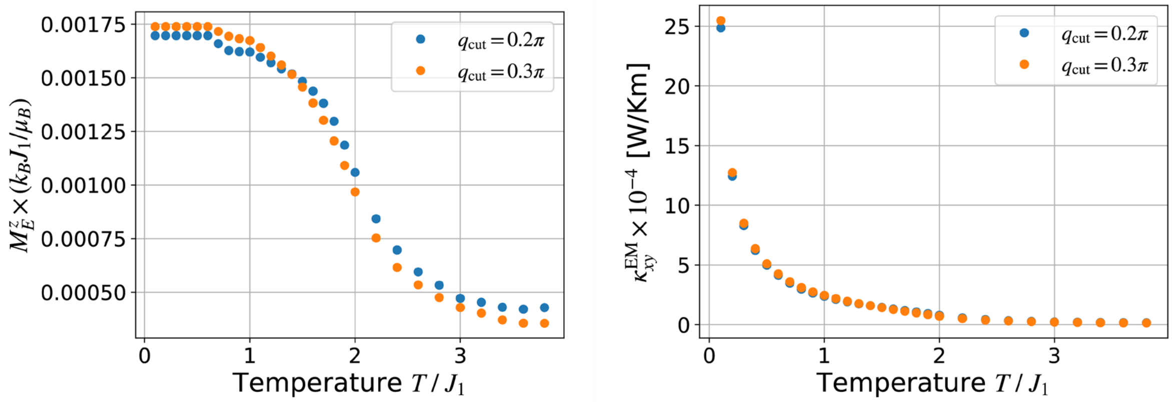

Supplementary Fig. 7 | Temperature dependence of $M^{E,z}$ and $\kappa_{xy}^{\rm EM}$. We assumed that $M^{E,z}$ is constant at $T\le 0.5J_1$, which follows from the fact that $\mu^{E,z}$ is constant in that region.

Supplementary Note 6: Stochastic Landau-Lifshitz-Gilbert equation

The Kubo term $\kappa_{ij}^{\rm Kubo}$ can be calculated by Supplementary Eq. 4, which requires the real-time

evolution of spins. This is numerically realized by solving the Landau-Lifshitz-Gilbert (LLG) equation with stochastic force. The LLG equation, which describes the equation of motion of magnetic moments $\mathbf{M}_i$, reads

$$\frac{d\mathbf{M}_i}{dt} = -\gamma \mathbf{M}_i \times \mathbf{B}_i^{\mathrm{eff}} - \frac{\alpha\gamma}{M} \mathbf{M}_i \times \left(\mathbf{M}_i \times \mathbf{B}_i^{\mathrm{eff}}\right), \tag{11}$$

where $\gamma = g\mu_{\mathrm{B}}/\hbar$ is the gyromagnetic ratio, $M = |\mathbf{M}|$ the saturation magnetization, α the dimensionless damping parameter, and $\mathbf{B}_i^{\mathrm{eff}}$ the effective magnetic field consists of the external magnetic field and the spin-spin interaction. We assume $\alpha = 0.001$ based on the experimental estimation in the Supplementary Reference[8]. The magnetic moment is related to spin by $\mathbf{M}_i = -\mu_{\mathrm{B}}\mathbf{S}_i$. By replacing t with the dimensionless time, $t \to t \times \mu_{\mathrm{B}}/(\gamma E_0)$ with $E_0 = 2.0$ meV being the value of J_1, Supplementary Eq. 11 can be rewritten in the dimensionless form:

$$d\mathbf{S}_i = -\frac{1}{1+\alpha^2} \mathbf{S}_i \times \left[\left(\mathbf{h}_i^{\mathrm{eff}} dt + \mathbf{h}_i^{\mathrm{fl}}(t)\right) + \alpha \mathbf{S}_i \times \left(\mathbf{h}_i^{\mathrm{eff}} dt + \mathbf{h}_i^{\mathrm{fl}}(t)\right)\right]. \tag{12}$$

Here, $\mathbf{h}_i^{\mathrm{eff}} = \mu_B \mathbf{B}_i^{\mathrm{eff}}$ is the effective Zeeman field, which is given by

$$\mathbf{h}_i^{\mathrm{eff}} = \mathbf{h} - \frac{\partial H_i}{\partial \mathbf{S}_i}. \tag{13}$$

The first term in Supplementary Eq. 13 is the external magnetic field, and the second term consists of the exchange and DM interactions. The random force $\mathbf{h}_i^{\mathrm{fl}}(t)$ satisfies

$$E\left[h_i^{\mathrm{fl},\mu}(t)\right] = 0, \qquad E\left[h_i^{\mathrm{fl},\mu}(t) h_j^{\mathrm{fl},\nu}(s)\right] = 2D\delta_{i,j}\delta^{\mu,\nu}\delta(t-s),$$

From the fluctuation-dissipation theorem, the thermal equilibrium is correctly reproduced by setting $D = \alpha T$. Here, $E[\cdots]$ denotes the statistical average. $\mathbf{h}_i^{\mathrm{fl}}(t)$ is given by

$$\mathbf{h}_i^{\mathrm{fl}}(t)dt = \sqrt{2D}\, d\mathbf{W}_i(t),$$

where $\mathbf{W}_i^{\mu}(t)$ is the Wiener process and $d\mathbf{W}_i^{\mu}(t) = \mathcal{N}(0, dt)$ with $\mathcal{N}(0, dt)$ being the normal distribution with mean 0 and variance dt[9].

There are two methods to numerically integrate Supplementary Eq. 12:[9] the Heun method and the implicit midpoint method. The latter method has the advantage that the norm of spins is automatically preserved, while it is not guaranteed in the Heun method. On the other hand, the implicit method requires more computational time, and therefore, we adopted the Heun method. Here, we symbolically write the LLG equation as $dS(t) = dF\big(S(t), W(t)\big)$, where $S(t)$ indicates spins and $W(t)$ the random force. Then the Heun method updates $S(t)$ by

$$S(t+dt) - S(t) = \frac{1}{2}\left[dF\big(S(t), W(t)\big) + dF\left(\tilde{S}(t+\delta t), W(t+\delta t)\right)\right],$$

$$\tilde{S}(t+dt) \coloneqq S(t) + dF\big(S(t), W(t)\big).$$

Since the norm of spins is not necessarily preserved, we normalize the spin lengths at each update.

Supplementary Note 7: Calculation of $\kappa_{xy}^{\mathrm{Kubo}}$

In our calculation, we first prepare the equilibrium state by the MC updates (with the same condition as the EM) and then update spins by Supplementary Eq. 12. The used time step is $dt = 0.002$ for $T < 1$ and $dt = 0.001$ for $T \geq 1$. The reason for using smaller dt for $T \geq 1$ is because the decay rate of the correlation functions becomes rapid at high temperatures. The energy current at each site and bond, $\mathbf{J}_i^E$ and $j_{i,j}^E$, are calculated for every 20 LLG updates. A large amount of data have to be collected to obtain convergent results for $C_{\mu\nu}(t) = \langle J^{E,\mu}(t) J^{E,\nu}(0) \rangle$, and therefore, we adopted the following strategy according to the Supplementary References[6,7]:

1. First, we prepare the MC-equilibrated state.
2. We perform $N_{\mathrm{loops}} = 2 \times 10^6$ LLG updates.
3. From the collected N_{loops} samples, the n-th to $n + N_t$-th data are grouped into 1 sample for $C_{\mu\nu}(t)$ $(0 \leq t \leq N_t dt)$. Thus we can extract $N_{\mathrm{loops}} - N_t$ sets of data in this sequence.
4. Then we prepare another equilibrium state by 100 MC sweeps.
5. Repeat step 3.
6. The above routine is done in 1000 parallel lattices.

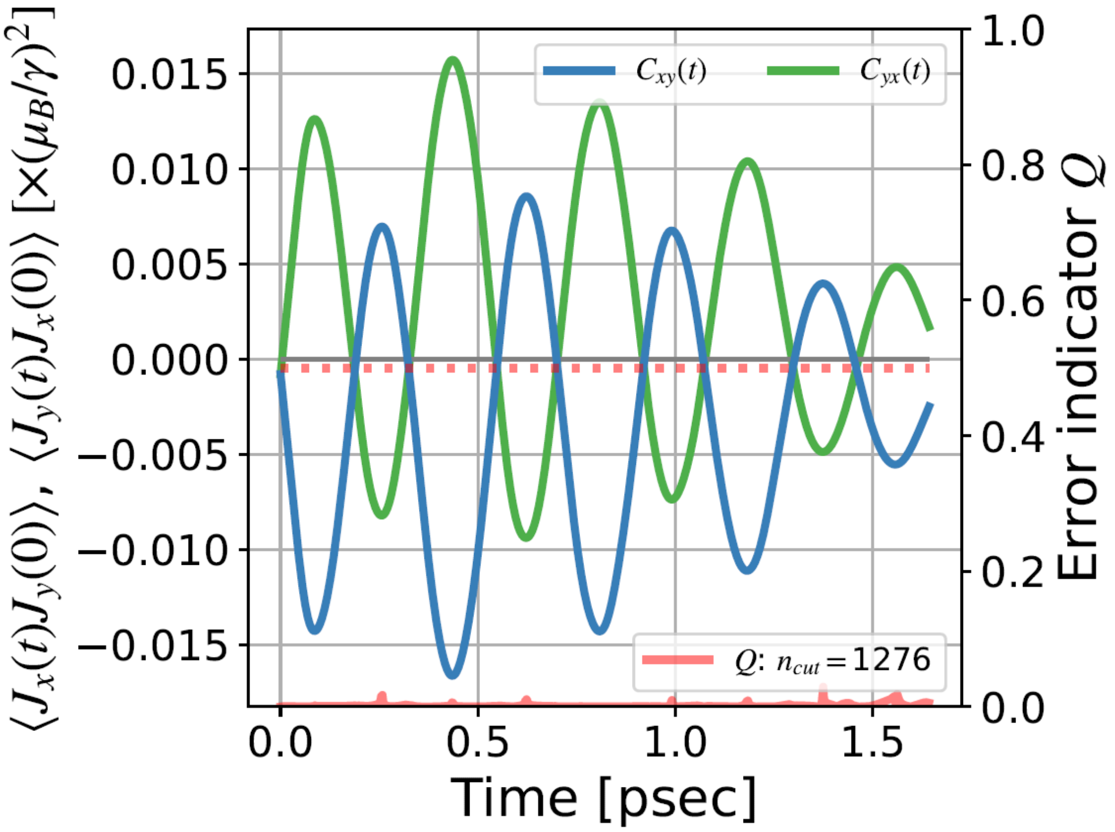

Supplementary Fig. 8 | Plot of $C_{xy}(t) = \langle J^{E,x}(t)J^{E,y}(t)\rangle$, $C_{yx}(t) = \langle J^{E,y}(t)J^{E,x}(0)\rangle$ and the error indicator $Q(t)$ at $T = J_1$. The horizontal red line is the threshold to determine the cutoff time: $Q(t) < 0.5$.

Supplementary Fig. 8 shows the time profile of $C_{xy}(t)$ and $C_{yx}(t)$. There are two relationships connecting C_{xy} and C_{yx}; one is from the Onsager's reciprocal relationship, $\kappa_{xy}(h) = \kappa_{yx}(-h)$. Typically, the (thermal) Hall conductance is antisymmetric about h, and hence $\kappa_{xy}(h) = -\kappa_{yx}(h)$, from which we expect

$$C_{xy}(t) \overset{!}{=} - C_{yx}(t). \tag{14}$$

The other is $C_{xy}(t) = \langle J^{E,x}(t)J^{E,y}(0)\rangle = \langle J^{E,y}(0)J^{E,x}(t)\rangle = C_{yx}(-t)$, because all observables are commutable in the classical simulation. In particular, we have $C_{xy}(t=0) = C_{yx}(t=0)$. In the actual simulation, Supplementary Eq. 14 is not guaranteed due to numerical errors. Mook *et al.*[6] introduced an error indicator for C_{xy} and C_{yx}, which measures the discrepancy between the two:

$$Q(t) := \frac{1}{4}\left| E\left(\frac{\dot{C}_{xy}(t)}{C_{xy}(t)}, \frac{\dot{C}_{yx}(t)}{C_{yx}(t)}\right) + E\left(\dot{C}_{xy}(t), \dot{C}_{yx}(t)\right) + E\left(C_{xy}(t), C_{yx}(t)\right) - 1 \right|, \qquad (15)$$

where $E(x, y) := 1 - \frac{2xy}{x^2+y^2} = \frac{(x-y)^2}{x^2+y^2}$. This measure of error is shown by red bars in Supplementary Fig. 8. $Q(t)$ ranges from 0 to 1, and the smaller $Q(t)$ is, the less numerical error is. $Q(t)$ tends to be enhanced at a long time t, and the data at that time is less accurate than shorter time.

From Supplementary Eq. 4, $\kappa_{xy}^{\mathrm{Kubo}}$ is obtained via the integral over time t, which is difficult in numerics due to numerical errors at long t. There are two possible methods to perform the time integral:

1. Directly integrate the raw data of $\tilde{C}_{xy}(t)$, or
2. Identify the fitting function of $\tilde{C}_{xy}(t)$ and analytically integrate it.

In any case, we have to introduce some cutoff time t_{cut} for integral in the case of 1., and for the fitting in the case of 2. above. Here, we assume a threshold value of 0.5 for $Q(t)$, and drop the data with $Q(t) \geq 0.5$. Thus, t_{cut} is defined as the largest t which satisfies $Q(t) < 0.5$.

Based on the cutoff, we fit the data within $0 \leq t \leq t_{\mathrm{cut}}$ by the linear combination of damped oscillators (DOs)[7]:

$$F_{xy;\mathrm{fit}}(t) = \sum_{n=1}^{N_{\mathrm{mode}}} A_n e^{-b_n t} \sin(\omega_n t + \phi_n). \qquad (16)$$

Here, we define t_{cut} as the minimum time which satisfies $Q(t) < 0.5$. Since Supplementary Eq. 14 should hold ideally, we anti-symmetrize C_{xy}; we use $\tilde{C}_{xy}(t) := \left(C_{xy}(t) - C_{yx}(t)\right)/2$ instead of $C_{xy}(t)$ itself. For $T \gtrsim 0.3J_1$, $C_{xy}(t)$ is well fitted by 2 DOs, while 3 DOs should be used for fitting at higher temperatures (see Supplementary Fig. 9). As an indicator of the accuracy of the fitting, we used the coefficient of determination R^2. For M samples $\{y_n\}_{n=1,\cdots,M}$, whose mean value is $\bar{y} = \frac{1}{M}\sum_{n=1}^{M} y_n$, and the fitted data y_{fit}, R^2 is defined by

$$R^2 = 1 - \frac{\sum_{n=1}^{M}(y_n - y_{\mathrm{fit}})^2}{\sum_{n=1}^{M}(y_n - \bar{y})^2}.$$

R^2 ranges from 0 to 1, and the closer to 1 R^2 is, the more accurate the fitting is.

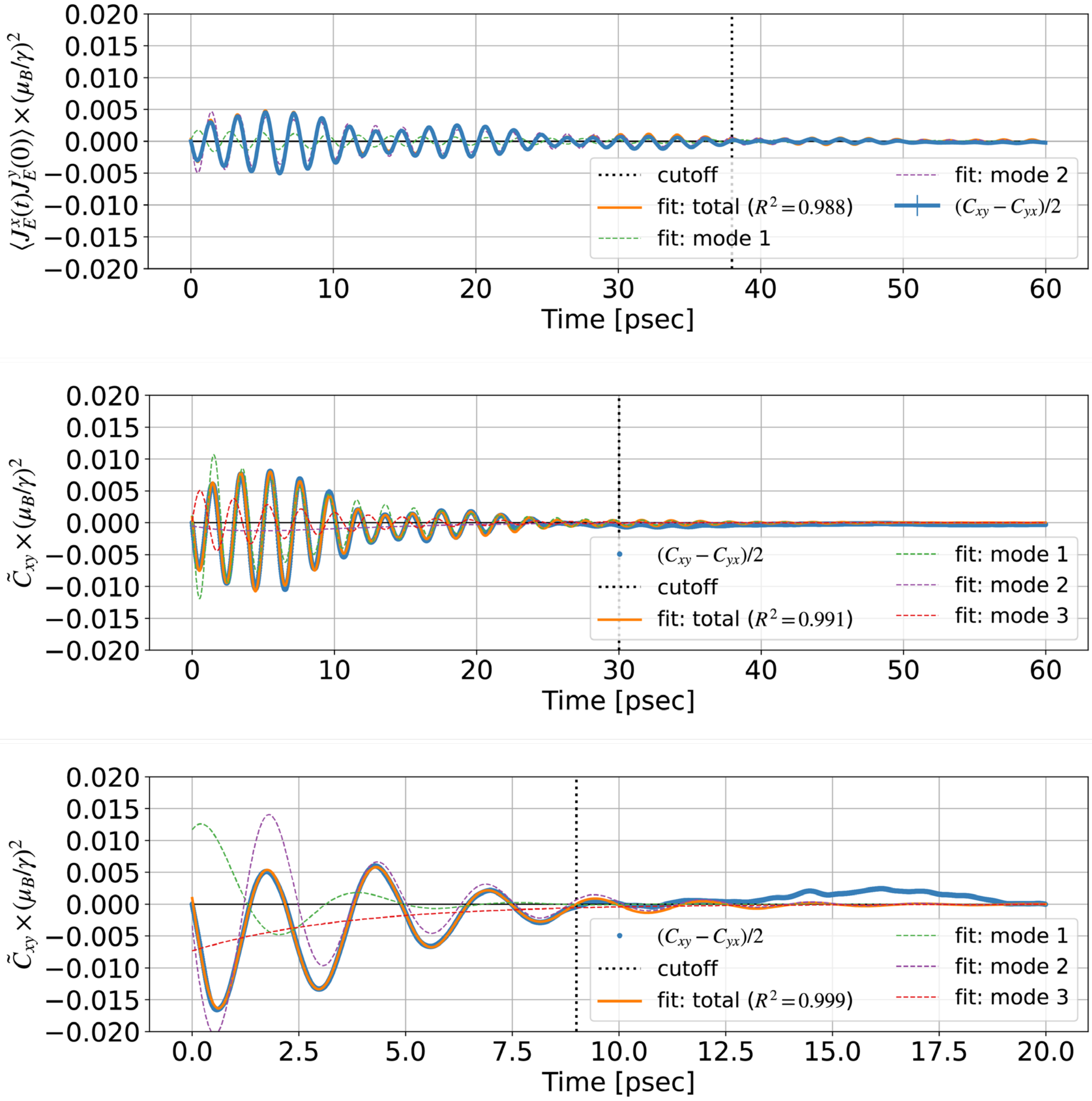

Supplementary Fig. 9 | Fitting of $\tilde{C}_{xy}(t)$, anti-symmetrized correlation function, at $T/J_1 = 0.2$, 0.5, and 1.5. $C_{xy}(t)$ is fitted by the combination of two (three) DOs for $T/J_1 < 0.5 (\geq 0.5)$. As T increases, the amplitude of $\tilde{C}_{xy}$ is enhanced while the decay rate becomes more rapid. R^2 is the coefficient of determination.

The number of DOs for fitting is a subtle problem because $\tilde{C}_{xy}(t)$ is a nonlinear function with many parameters and hence a slight change of t_{cut}, numerical errors, etc., could significantly affect the fitting, and $\kappa_{xy}^{\mathrm{Kubo}}$ is very sensitive to the slight change in the fitting. Therefore, we tried three methods: (i) fitting with 2 DOs, (ii) fitting with 3 DOs and (iii) numerical integral and compared those results (Figure S10). The three methods are consistent in $T \geq J_1$, while

they are unstable in $T < J_1$.

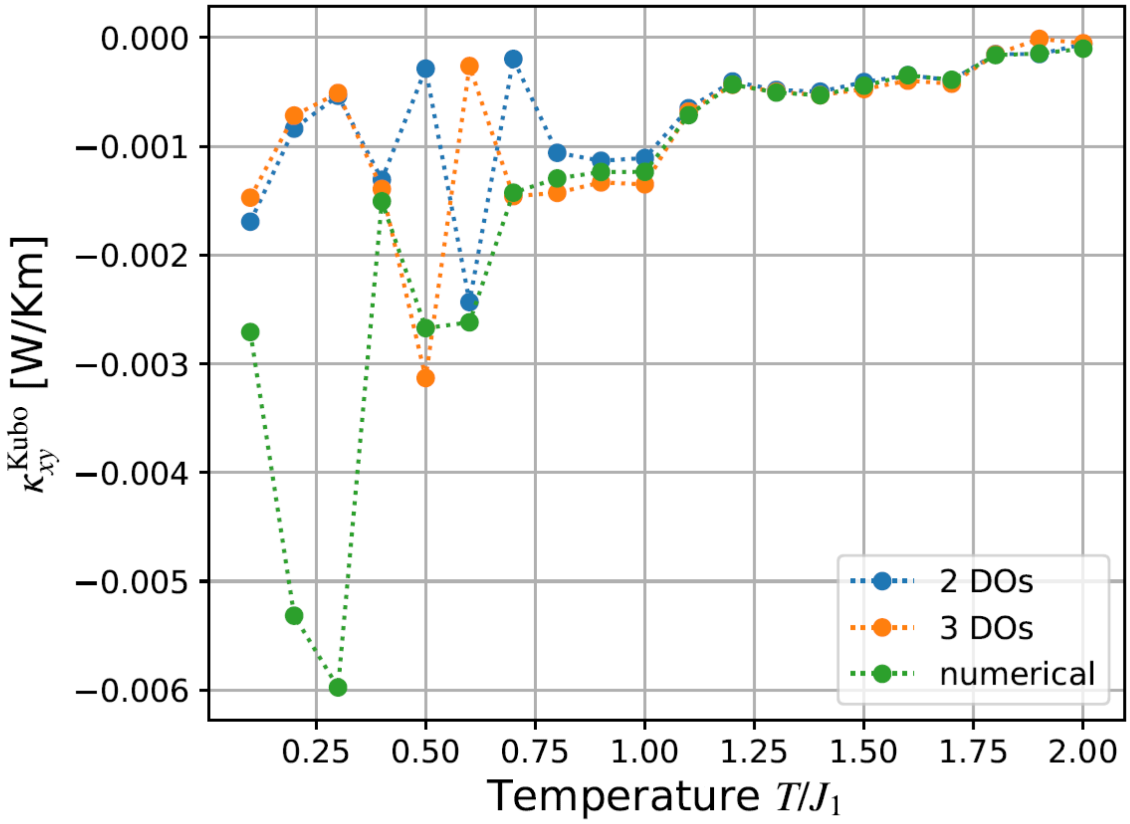

Supplementary Fig. 10 | Temperature dependence of $\kappa_{xy}^{\mathrm{Kubo}}$ calculated by different methods. The blue and orange lines represent the fitting results with two and three-damped oscillators. The green line shows the result of the numerical integral.

Supplementary Note 8: Comparison with the spin-spin correlation

From Fig. 3 in the main text, one can see that κ_{xy} approaches to nearly zero near T_N. On the other hand, the magnetic correlation length of $YMnO_3$, estimated from the neutron powder diffraction, persists up to a much higher temperature[2]. Naively, the short-range spin-spin correlation carries heat, which contradicts our calculational result. We examined the spin-spin correlation function in our classical model to elucidate this point. Then we concluded that the correlation between spins is largely suppressed at $\mathbf{q}$ with high energy, which is consistent with the suppression of the thermal Hall coefficient.

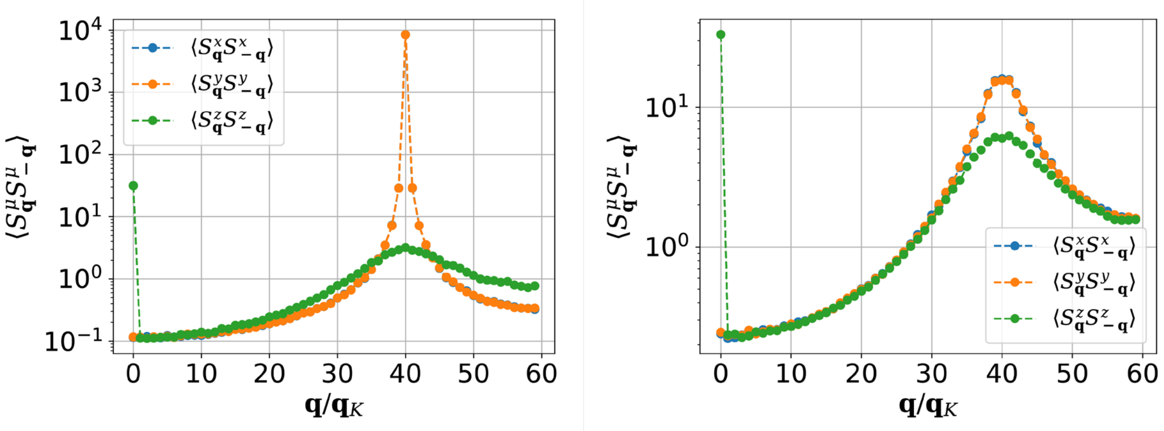

Supplementary Fig. 11 | The equal-time spin-spin correlation function. The horizontal axis is taken along the line connecting $\mathbf{q} = (0,0)$ and $\mathbf{q} = \mathbf{q}_K$.

Supplementary Fig. 11 shows the equal-time spin-spin correlation function $\langle S^\mu_{\mathbf{q}} S^\mu_{-\mathbf{q}} \rangle$ $(\mu = x, y, z)$ at $T/J_1 = 1.2$ and 2.4. It has a peak structure at $\mathbf{q}_K = \left(\frac{2\pi}{3}, \frac{2\pi}{\sqrt{3}}\right)$, which corresponds to the K point of the original triangular lattice (i.e., without trimerization). Below T_N, $\langle S^\mu_{\mathbf{q}} S^\mu_{-\mathbf{q}} \rangle$ has a sharp, δ-function-like structure, while it is softened above T_N. Since the Zeeman field is acting on the spins, $\langle S^z_{\mathbf{q}} S^z_{-\mathbf{q}} \rangle$ has another peak at $\mathbf{q} = (0,0)$. The peak height and the full width at half maximum (FWHM) are shown in Supplementary Fig. 12 as a function of T, from which one can convince that the softening of the peak structure happens at $T = T_N$.

The real-time profile of $\langle S^\mu_{\mathbf{q}}(t) S^\mu_{-\mathbf{q}}(0) \rangle$ at $\mathbf{q} = \mathbf{q}_K$ is shown in Supplementary Fig. 13. The in-plane components ($\langle S^x_{\mathbf{q}_K}(t) S^x_{-\mathbf{q}}(0) \rangle$ and $\langle S^y_{\mathbf{q}}(t) S^y_{-\mathbf{q}_K}(0) \rangle$) and the out-of-plane component $\langle S^z_{\mathbf{q}_K}(t) S^z_{-\mathbf{q}_K}(0) \rangle$ have different behaviours of the time-dependence. $\langle S^x_{\mathbf{q}_K}(t) S^x_{-\mathbf{q}_K}(0) \rangle$ and $\langle S^y_{\mathbf{q}_K}(t) S^y_{-\mathbf{q}_K}(0) \rangle$ monotonically decay as t increases. In contrast, $\langle S^z_{\mathbf{q}_K}(t) S^z_{-\mathbf{q}_K}(0) \rangle$ oscillates while decaying, and the decay rate is faster than the in-plane components. The slow decay of the in-plane components reflects the gapless mode related to the U(1) symmetry. One can estimate the times at which $\langle S^z_{\mathbf{q}}(t) S^z_{-\mathbf{q}}(0) \rangle$ vanishes to be ≈ 4, 1 and 0.5 psec for $T/J_1 = 1$, 2, and 3, respectively. One can estimate that the decay rate is approximately proportional to T^2. Also, the energy spectrum obtained from the Fourier transform of $\langle S^\mu_{\mathbf{q}}(t) S^\mu_{-\mathbf{q}}(0) \rangle$ are shown in Supplementary Fig. 14, consistent with the spin-wave analysis in the supplementary Reference[10].

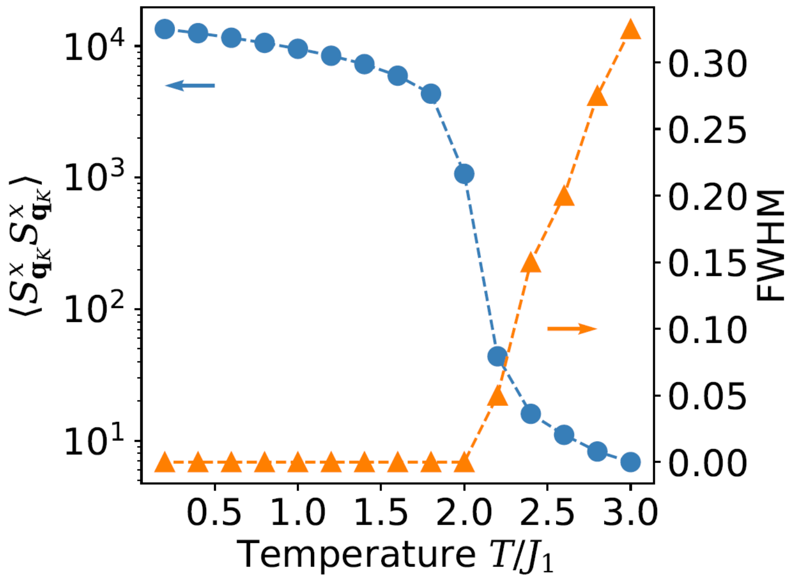

Supplementary Fig. 12 | The peak value and the FWHM of the equal-time spin-spin correlation function as a function of T.

As a comparison, we show the longitudinal and transverse components of the energy-current correlation function $\langle J^{E,\mu}(t)J^{E,\nu}(0)\rangle$ in Supplementary Fig. 15. In the right panel in Supplementary Fig. 15, the decaying behaviour of $\langle J^{E,x}(t)J^{E,x}(0)\rangle$ is shown, which is similar to that of $\langle S^z_{\mathbf{q}}(t)S^z_{-\mathbf{q}}(0)\rangle$. The times at which $\langle J^{E,x}(t)J^{E,x}(0)\rangle$ and $\langle J^{E,x}(t)J^{E,y}(0)\rangle$ vanish are almost identical to those of $\langle S^z_{\mathbf{q}}(t)S^z_{-\mathbf{q}}(0)\rangle$, about which there is no difference between the longitudinal and transverse components. The magnitude of $\langle J^{E,x}(t)J^{E,y}(0)\rangle$ is less than 100 times smaller than $\langle J^{E,x}(t)J^{E,x}(0)\rangle$, and therefore more vulnerable to thermal noise. $\langle J^{E,x}(t)J^{E,y}(0)\rangle$ at $T/J_1 = 3$ drops faster than the oscillation period and can no longer be fitted by the damped oscillator nor numerically integrated. As a result, $\kappa_{xy}^{\mathrm{Kubo}}$ is nearly zero at $T/J_1 = 3$.

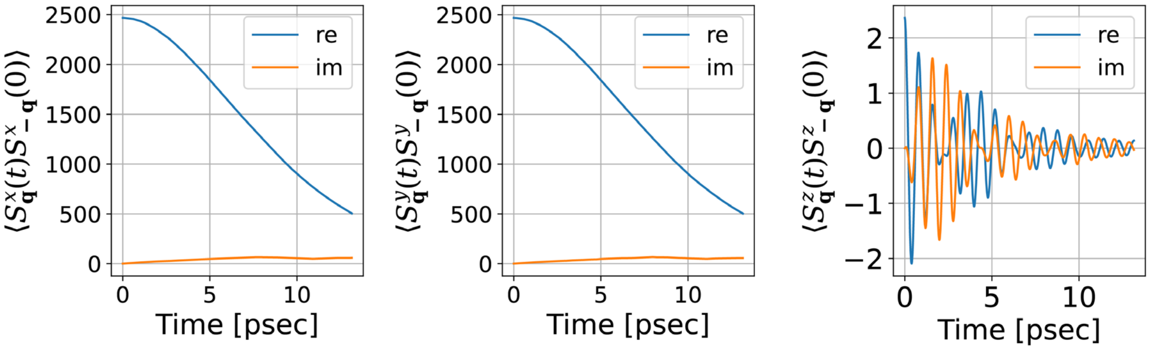

(a) $T\,/\,J_1 = 1$

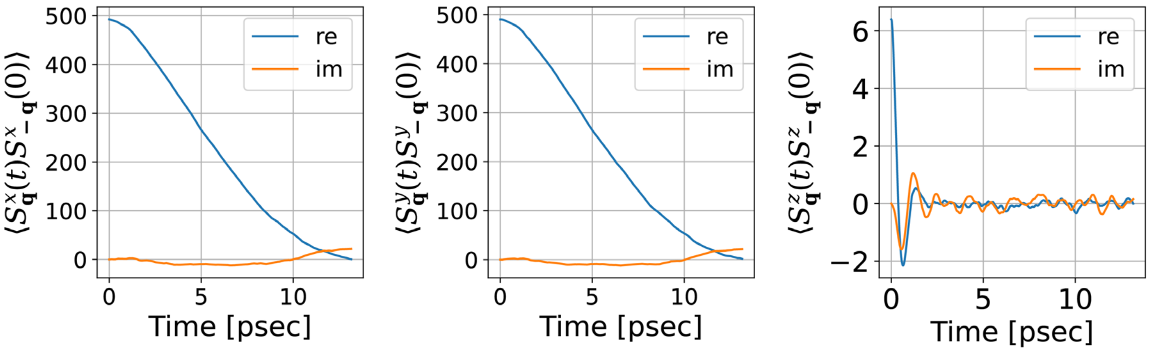

(b) $T\,/\,J_1 = 2$

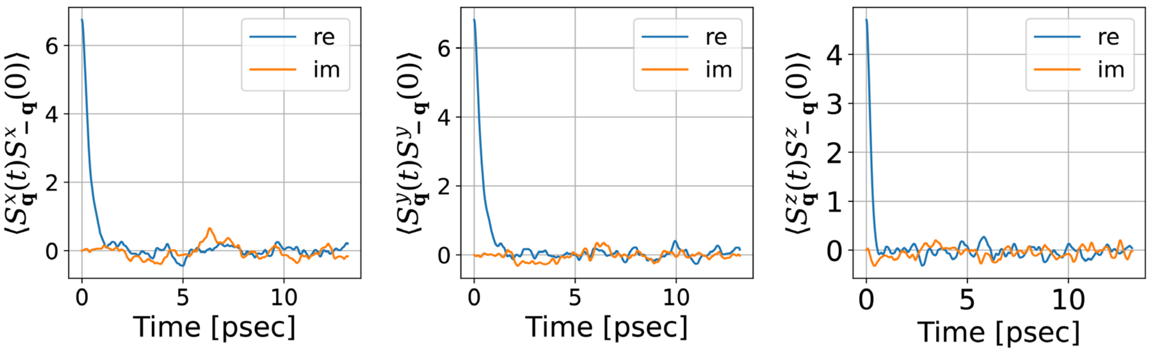

(c) $T\,/\,J_1 = 3$

Supplementary Fig. 13 | The real-time profile of the spin-spin correlation functions at $\mathbf{q} = \mathbf{q}_k$ and $T/J_1 = 1$, 2 and 3.

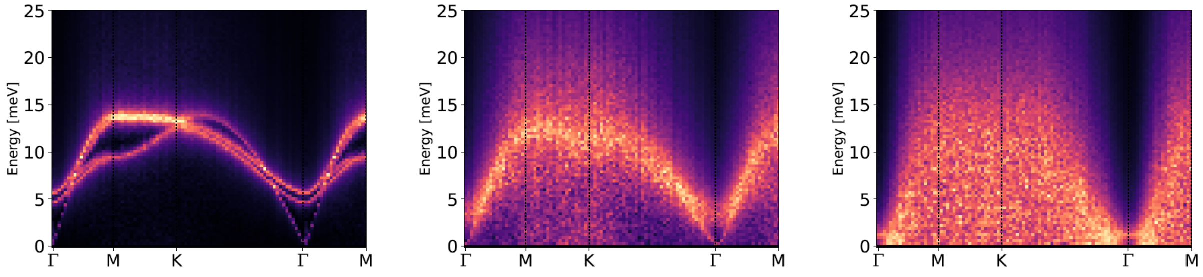

(a) $T/J_1 = 1$ (b) $T/J_1 = 2$ (c) $T/J_1 = 3$

Supplementary Fig. 14 | The energy spectrum of spins is obtained from the spin-spin correlation function.

(a) $T/J_1 = 1$

(b) $T/J_1 = 2$

(c) $T/J_1 = 3$

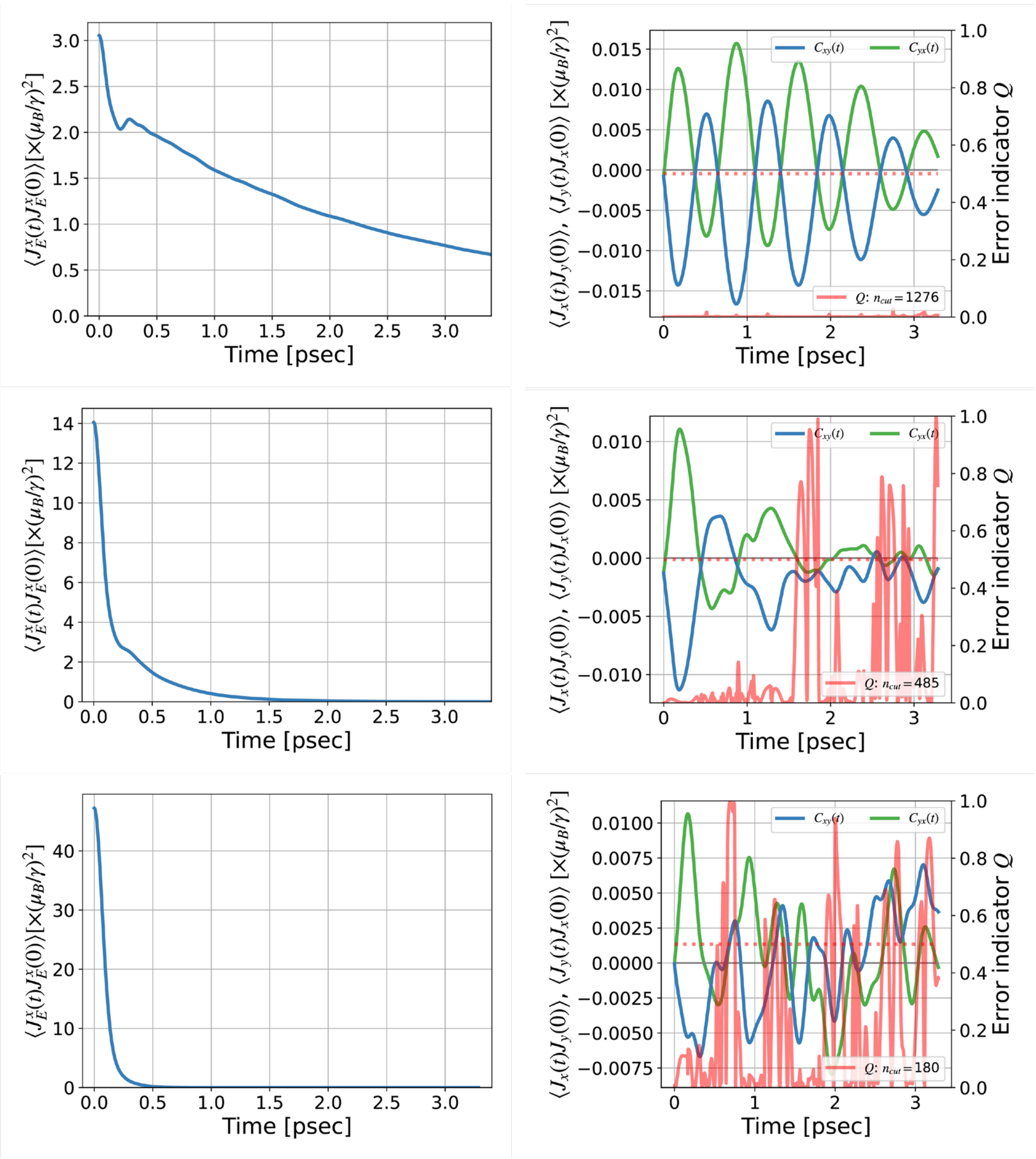

Supplementary Fig. 15 | The real-time profiles of the longitudinal and transverse energy-

current correlation functions at $T/J_1 = 1$, 2 and 3. The left and right panels show $\langle J^{E,x}(t)J^{E,x}(0)\rangle$ and $\langle J^{E,x}(t)J^{E,y}(0)\rangle$, respectively.

Supplementary Note 9: Contribution from phonons due to the Raman interaction

As a possible mechanism of the thermal Hall effect, we considered a contribution from the coupling of magnetization and phonons. We assumed a two-dimensional tight-binding model with only the nearest-neighboring bond couplings. The parameters are set to give rise to the transverse phonon velocity $v_T = 3000$ m/s[11]. The Raman interaction is given by $V = \lambda\sum_{\langle i,j\rangle}\mathbf{M}\times(\mathbf{u}_i - \mathbf{u}_j)\cdot\mathbf{P}_i$, where λ is the coupling constant and $\mathbf{M} = (0,0,M^z)$ is magnetization[12]. We take the value of $M^z(T)$ at B = 9 T observed experimentally[13]. Here we take the estimated value of the spin-phonon Raman interaction $\lambda\sim$ 0.005 meV from the phonon splitting in CrI_3 as the typical value of 3d systems[14].

The phonon Hamiltonian is given by $H_{\text{ph}} = \frac{1}{2}\sum_k \begin{bmatrix} u_k^x \\ u_k^y \\ P_k^x \\ P_k^y \end{bmatrix}^T \begin{bmatrix} D_k & -A_k \\ A_k & 1/m \end{bmatrix} \begin{bmatrix} u_k^x \\ u_k^y \\ P_k^x \\ P_k^y \end{bmatrix}$, with D_k the dynamical matrix, m mass of Mn^{3+} ions, and $A_k = \lambda M^z \sum_{n=1}^{3}(1-\cos kd_n)\begin{bmatrix} 0 & -1 \\ 1 & 0 \end{bmatrix}$. Each component of D_k is given by $D_k^{xx} = \frac{c}{2}\left(3 - \cos k_x - \cos\frac{k_x}{2}\cos\frac{\sqrt{3}k_y}{2}\right)$, $D_k^{yy} = \frac{3c}{2}\left(1 - \cos\frac{k_x}{2}\cos\frac{\sqrt{3}k_y}{2}\right)$ and $D_k^{xy} = D_k^{yx} = \frac{c}{2}\sin\frac{k_x}{2}\sin\frac{\sqrt{3}k_y}{2}$. Here, c is the spring constant, which is related to the transverse phonon velocity by $v_T = \frac{\sqrt{3}}{4a}\sqrt{\frac{c}{m}}$ (a is the lattice constant). By introducing a unitary transformation $\begin{bmatrix} u_k^x \\ u_k^y \\ P_k^x \\ P_k^y \end{bmatrix} = \left(\frac{1}{\sqrt{2}}\begin{bmatrix} 1 & 1 \\ -i & i \end{bmatrix}\otimes\begin{bmatrix} 1 & 0 \\ 0 & 1 \end{bmatrix}\right)\begin{bmatrix} \alpha_k \\ \beta_k \\ \alpha_{-k}^\dagger \\ \beta_{-k}^\dagger \end{bmatrix}$, we can construct a BdG-type Hamiltonian, and the Berry curvature $\Omega_{\mathbf{k},n}^z$ can be defined (n=1,2,3 is index for the eigenmodes). The thermal Hall conductivity κ_{xy} is given by[15]

$$\kappa_{xy} = \frac{-k_B^2 T}{\hbar V l_c}\sum_{\mathbf{k}}\sum_{n=1}^{3}\sum_{\sigma=\uparrow,\downarrow}\left[c_2\left(n_B(\omega_{\mathbf{k},n,\sigma})\right) - \frac{\pi^2}{3}\right]\Omega_{\mathbf{k},n,\sigma}^z, \tag{17}$$

where n_B is the Bose-Einstein distribution function and $c_2(x) = (1+x)\left(\ln\frac{1+x}{x}\right)^2 - (\ln x)^2 - 2Li_2(-x)$. The two-dimensional conductivity is translated to a three-dimensional one by dividing by the inter-layer distance $l_c = 5.7$ Å.

The intrinsic contribution to the thermal Hall conductivity is shown in Supplementary Fig. 16,

which is around ~ - 3 x10^{-5} W K^{-1} m^{-1} at 100 K and one order of magnitude smaller than that from spins in this low-temperature region.

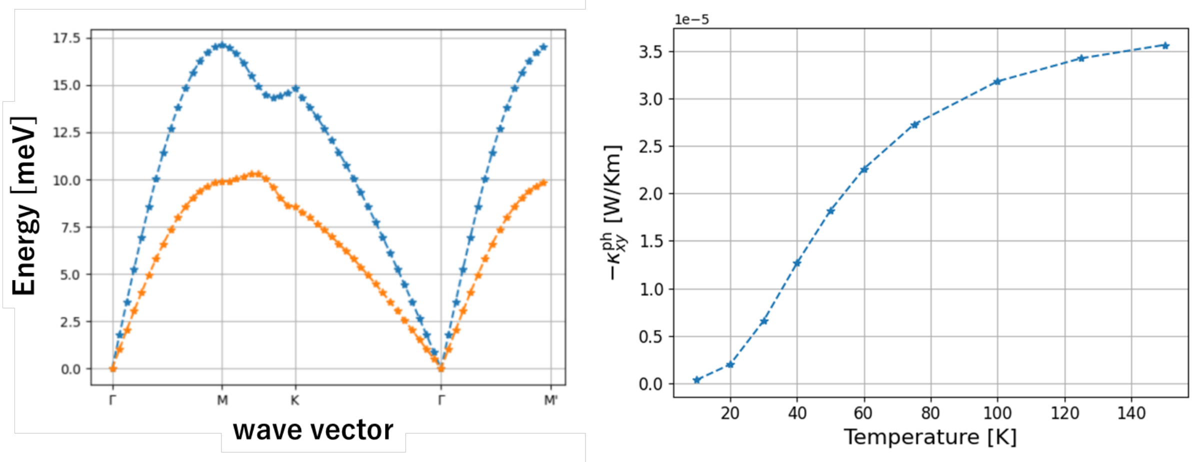

Supplementary Fig. 16 | The phonon dispersion (left) and its thermal Hall conductivity (right) of the model calculation for $YMnO_3$. The intrinsic contribution to the thermal Hall conductivity is smaller than that from spins in this low-temperature region.

Supplementary Note 10: Theoretical calculation of magnetic susceptibility and scalar spin chirality

In order to see the topological nature of the spin fluctuation, we have theoretically calculated the uniform magnetic susceptibility with and without the DM interaction, as shown in Supplementary Fig. 17a below. Above T_N we find the Curie-Weiss (CW) behavior with negative CW temperature, while it deviates from CW behavior from slightly above T_N.

The effect of DM interaction is mainly the shift of T_N. Instead of the magnetic susceptibility, we can directly calculate the temperature dependence of the uniform scalar spin chirality in this trimerized triangular lattice, which is shown in Supplementary Fig. 17b.

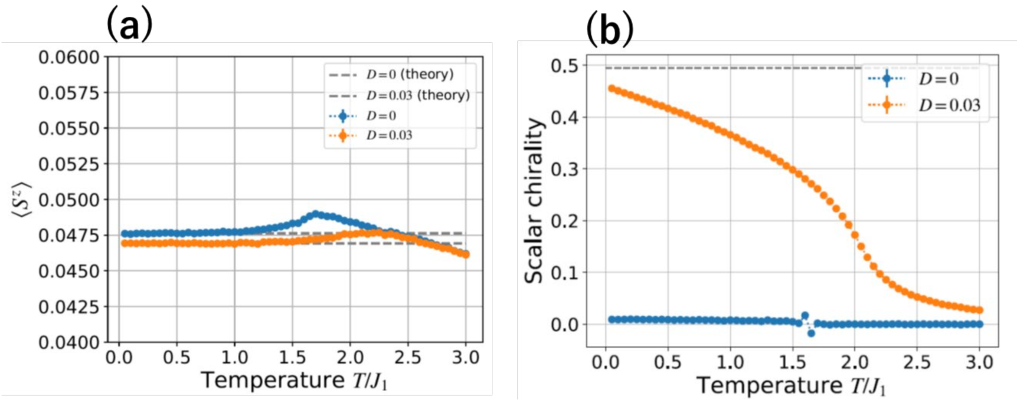

Supplementary Fig. 17 | It shows the Temperature dependence of (a) the uniform magnetic susceptibility and (b) the scalar spin chirality of the Heisenberg model on a trimerized triangular lattice. The parameters of the model are the same as in the main text. The magnetic field is B = 9 T both in panels (a) and (b)."

Supplementary References

1. Kim, H.-L. *et al.* Modular thermal Hall effect measurement setup for fast-turnaround screening of materials over wide temperature range using capacitive thermometry. *Rev. Sci. Instrum.* **90**, 103904 (2019).
2. Sharma, P. A. *et al.* Thermal Conductivity of Geometrically Frustrated, Ferroelectric YMnO3: Extraordinary Spin-Phonon Interaction. *Phys. Rev. Lett.* **93**, 177202 (2004).
3. Sologubenko, A. V., Giannó, K., Ott, H. R., Ammerahl, U. & Revcolevschi, A. Thermal Conductivity of the Hole-Doped Spin Ladder System Sr14-xCaxCu24O41. *Phys. Rev. Lett.* **84**, 2714–2717 (2000).
4. Qin, T., Zhou, J. & Shi, J. Berry curvature and the phonon Hall effect. *Phys. Rev. B* **86**, 104305 (2012).
5. Kapustin, A. & Spodyneiko, L. Absence of Energy Currents in an Equilibrium State and Chiral Anomalies. *Phys. Rev. Lett.* **123**, 060601 (2019).
6. Mook, A., Henk, J. & Mertig, I. Spin dynamics simulations of topological magnon insulators: From transverse current correlation functions to the family of magnon Hall effects. *Phys. Rev. B* **94**, 174444 (2016).
7. Carnahan, C., Zhang, Y. & Xiao, D. Thermal Hall effect of chiral spin fluctuations. *Phys. Rev. B* **103**, 224419 (2021).
8. Tzschaschel, C., Satoh, T. & Fiebig, M. Efficient spin excitation via ultrafast damping-like torques in antiferromagnets. *Nat. Commun.* **11**, 6142 (2020).
9. d'Aquino, M., Serpico, C., Coppola, G., Mayergoyz, I. D. & Bertotti, G. Midpoint numerical technique for stochastic Landau-Lifshitz-Gilbert dynamics. *J. Appl. Phys.* **99**, 08B905 (2006).
10. Kim, K.-S., Lee, K. H., Chung, S. B. & Park, J.-G. Magnon topology and thermal Hall effect in trimerized triangular lattice antiferromagnet. *Phys. Rev. B* **100**, 064412 (2019).
11. Rushchanskii, K. Z. & Ležaić, M. Ab Initio Phonon Structure of h -YMnO 3 in Low-Symmetry Ferroelectric Phase. *Ferroelectrics* **426**, 90–96 (2012).
12. Sheng, L., Sheng, D. N. & Ting, C. S. Theory of the Phonon Hall Effect in Paramagnetic Dielectrics. *Phys. Rev. Lett.* **96**, 155901 (2006).
13. Muñoz, A. *et al.* Magnetic structure of hexagonal RMnO3 (R=Y,Sc): Thermal evolution from neutron powder diffraction data. *Phys. Rev. B* **62**, 9498–9510 (2000).
14. Bonini, J. *et al.* Frequency Splitting of Chiral Phonons from Broken Time-Reversal Symmetry in CrI3. *Phys. Rev. Lett.* **130**, 086701 (2023).
15. Matsumoto, R., Shindou, R. & Murakami, S. Thermal Hall effect of magnons in magnets with dipolar interaction. *Phys. Rev. B* **89**, 054420 (2014).
